# Crystalline orientation control of the platelet Nd_2_Fe_14_B phase to produce magnetic anisotropy via electromagnetic vibration processing

**DOI:** 10.1038/s41598-019-42053-9

**Published:** 2019-04-05

**Authors:** Mingjun Li, Takuya Tamura

**Affiliations:** National Institute of Advanced Industrial Science and Technology (AIST), Magnetic Powder Metallurgy Research Center, 2266-98, Moriyama, Nagoya, 463-8560 Japan

## Abstract

Controlled crystalline orientation of the discontinuous phase in a composite enables the production of improved anisotropic properties, e.g., well-aligned Nd_2_Fe_14_B platelets by hot pressing and then soaking in a low-melting Nd-Cu eutectic melt to infiltrate to grain boundary. Alternatively, an anisotropic magnet can be fabricated by sintering Nd_2_Fe_14_B powder pre-aligned with a static magnetic field. In this study, we used a two-step electromagnetic vibration (EMV) technique to solidify the Nd_70_Cu_30_-30wt% Nd_2_Fe_14_B alloy, by which the magnetic Nd_2_Fe_14_B compound could be segmented into short laths and the easy magnetisation axes of these discontinuous platelets could be highly aligned, as revealed by electron backscatter diffraction (EBSD) patterns. Magnetic properties showed that the alloy exhibited strong anisotropy in its magnetism. Our present results opened a new avenue for the simple production of anisotropic Nd_2_Fe_14_B magnets via solidification without the powder metallurgy routine. Moreover, the technique is highly expected to be applied to other systems, e.g., graphene-reinforced metallic and/or polymer composites in which the alignment of graphene can maximise the anisotropy in the thermal or electrical properties of the composites.

## Introduction

The anisotropy of the Nd_2_Fe_14_B phase originates from the lattice symmetry of the intermetallic compound that has a tetragonal structure, in which each lattice cell contains four Nd_2_Fe_14_B formula units with 68 atoms (space group *P*4_2_/*mnm*)^1^. Further analysis revealed that each Nd_2_Fe_14_B unit cell consists of an eight-layer structure that is perpendicular to the *c* axis, making the atomic stacking structure along the *c* axis more complicated than that along the *a* axis in terms of the atomic sites, occupancies, and coordinates^2^.

The complicated atomic structure along the *c* axis makes the stacking of atoms more sluggish than that along the *a* axis upon solidification when considering the interface attachment kinetics^3,4^. This implies that the crystal-growth velocity along the *c* axis is more sluggish than that along the *a* axis, as the driving force for the crystal growth of an effective Nd_2_Fe_14_B micro-nucleus should be similar for different growth directions. Hence, a platelet morphology of macro Nd_2_Fe_14_B crystals with a high-aspect ratio is always preferred, and thus the crystal size along the *c* axis is much shorter than that along the *a* axis. This should be why Nd_2_Fe_14_B crystals^5,6^ and 3.4 at% Co and 0.6 at% Ga-doped Nd_2_Fe_14_B crystals^7^ produced by melt spinning, exhibit platelet morphology.

The *c* axis of the magnetic platelet corresponds to the [001] direction and is the easy magnetisation axis. When the *c* axis of each platelet is well aligned, an anisotropic magnet will be fabricated^8,9^, which is expected to produce higher remanence.

The EMV technique was initially invented to produce grain-refined solidification structures in aluminium alloys^10,11^ and then was improved to refine pure metals^12^ and alloys^13^. It was recently applied during semi-continuous casting to achieve refined structures and improved mechanical properties^14^. In addition to structure refinement, Tamura *et al*.^15^ reported that EMV could suppress effective crystal nucleation and thus improve the glass formation ability of a bulk Mg_65_Cu_25_Y_10_ alloy.

In the present study, we explored a new application of the technique by employing the Nd_70_Cu_30_-30wt%Nd_2_Fe_14_B alloy as a model alloy, upon which EMV was produced when alternating current (AC) passed through the alloy. A suitable kit of processing parameters enables the crystalline orientation of Nd_2_Fe_14_B platelets to be highly aligned and thus to produce strong anisotropy in magnetism. The segmentation and orientation mechanisms were discussed, and the future application of the present technique was briefly outlined.

## Results

### Structures without EMV processing

**Figure 1:**
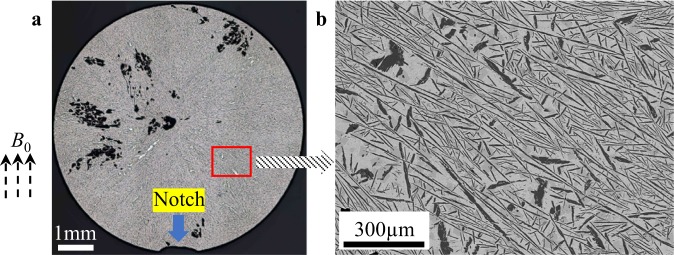
The structures of the alloy solidified in a static magnetic field without EMV processing. (**a**) The optical macro view of the cylindrical specimen solidified in a static magnetic field at *B*_0_ = 10 T exhibits a radiated structure from the centre spreading outside. (**b**) The scanning electron microscope (SEM) image depicts the structure highlighted by the red square in (**a**) with a developed feathery feature of the magnetic phase.

As the process involves both effects from the static magnetic field and the Lorentz force that follows Fleming’s left-hand rule, the specimen was first solidified from a semi-solid state in a static magnetic field of *B*_0_ = 10 T only and no AC was applied. The macro structure is similar to that of a cast ingot, as shown in Fig. 1a, which should be ascribed to the directional thermal release as the crystallisation heat is extracted by the metallic mould, and hence crystal growth commences from the mould wall and eventually encounters the rod centre, making the overall macro view exhibit a radiated pattern. Further observation reveals that the solidified structure consists of developed Nd_2_Fe_14_B platelets covering hundreds of micrometres in length, as revealed in Fig. 1b, indicating that the static magnetic field has little influence on the distribution and morphology of the magnetic compound that was produced in the metallic mould. Depiction of the structure is necessary, as it provides a comparable criterion to verify the validity of EMV processing in our subsequent presentation.

### Well-aligned Nd_2_Fe_14_B platelets in two-step EMV processing

**Figure 2:**
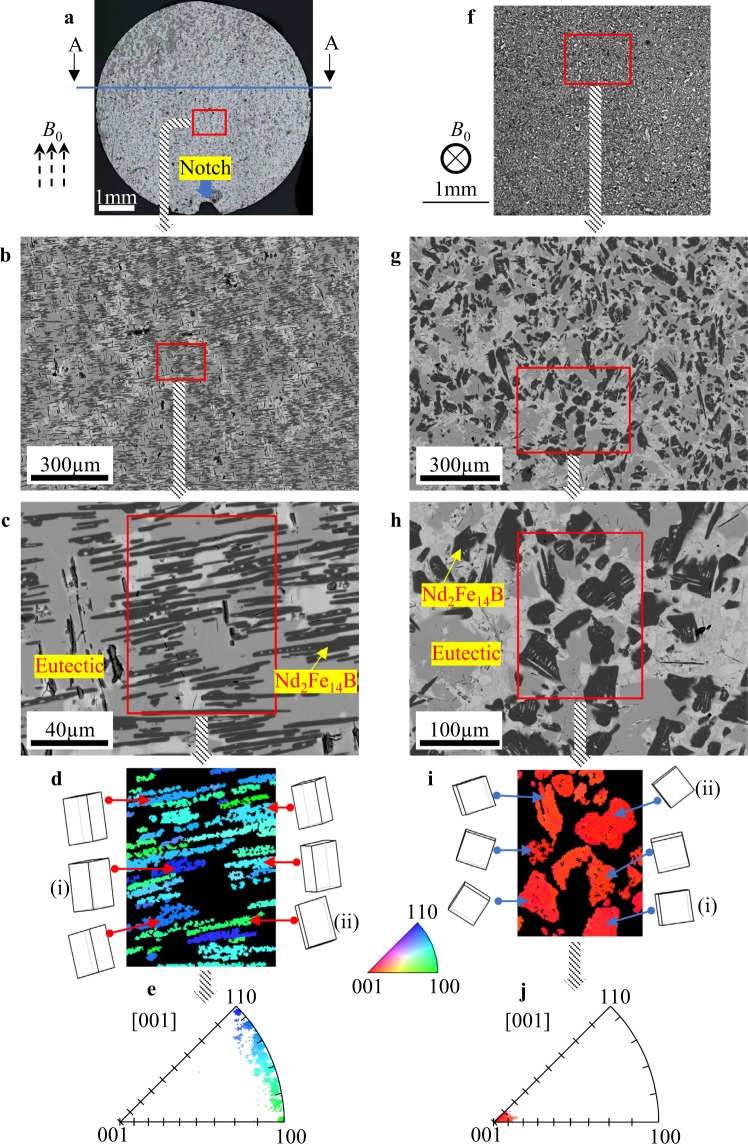
Well-aligned Nd_2_Fe_14_B platelets when two-step EMV processing was imposed. (**a**) The optical macro view of the specimen after two-step EMV processing shows the distribution of magnetic phase. (**b,c**) SEM images reveal structures with different magnifications. (**d**) The electron backscatter diffraction (EBSD) pattern depicts the crystalline orientation when the Nd_2_Fe_14_B phase is indexed in the red square in (**c**). The space lattices of six crystals are depicted, in which the deep-blue phase marked by (i) is near (110) and the green by (ii) is near (100). (**e**) The [001] IPF confirms the crystalline orientations of the magnetic phase biased towards (100) to (110). (**f**) The optical macro view of a sample shows the structure of the sectioning plane perpendicular to the direction of the static magnetic field, e.g., along the A-A direction in (**a**). (**g,h)** SEM images reveal morphologies with different magnifications. (**i**) The EBSD pattern depicts the crystalline orientation of Nd_2_Fe_14_B in the red square in (**h**). Space lattice of six crystals reveal that the planes indexed are (001). (**j**) The [001] IPF is highly intensified to (001).

When EMV is imposed at *f* = 250 Hz and then increased to *f* = 1000 Hz, i.e., two-step EMV processing, (see Methods for details), no radiated pattern can be discerned, and the overall structure becomes homogenous, as depicted in Fig. 2a. When the uniform area highlighted by a red square is magnified, the Nd_2_Fe_14_B platelets exhibit short and discontinuous bars instead of the long structures produced without EMV processing, as shown in Fig. 2b, indicating that the two-step EMV processing is effective in refining the Nd_2_Fe_14_B phase. Moreover, the striking feature is that all Nd_2_Fe_14_B platelets are well assembled with their longitudinal directions perpendicular to the static magnetic field. When the central area is further magnified, as revealed in Fig. 2c, one can find that except for a few long laths, most of platelets are on the order of tens of micrometres. The EBSD map of the red square region, together with the six space lattices in Fig. 2d, confirms that the crystal planes of these platelets range from (100) to (110) based on the adjacent colour triangle. Figure 2e shows the corresponding [001] inverse pole figure (IPF), revealing that the crystals projection concentrates from (100) to (110) within the band in less than 10 degrees, which once more demonstrates that their *c* axes should be highly oriented to [001] due to the tetragonal structure, as depicted by the space lattices.

When the sectioning plane is perpendicular to the magnetic field direction, it is inferred that *c* planes should be identified. Figure 2f shows the macro view observed in such a cutting plane with a homogenous distribution of the magnetic phase. In contrast to the well assembled slim and slight laths in Fig. 2b, the magnetic phase exhibits a block-like morphology without any trace of alignment at high magnification (Fig. 2g,h). The EBSD map exhibits a unique red colour, corresponding to (001). Six typical space lattices are depicted beside the map, showing their *c* planes. This is consistent with the deduction presented above. The [001] IPF indicates that magnetic phases are highly concentrated to (001) with their deviations less than 10 degrees.

When referring to the space lattice, one can readily see that the contour line of a crystal in Fig. 2i reveals the geometry of the crystal plane perpendicular to the *c* axis direction, i.e., the contour lines of (i) and (ii) may correspond to (100) and (110) planes with a deviation of a few degrees if captured perpendicular to the present observation plane. Note that the contour line of a crystal always covers from tens to a hundred of micrometres; it is consistent with the length scale in its longitude direction, as shown in Fig. 2c. Consider that the thickness of a platelet in Fig. 2c, is always in several micrometres along its *c* axis direction, the magnetic phase has a rather large aspect ratio with a typical platelet morphology.

### Randomly distributed Nd_2_Fe_14_B platelets at one-step EMV processing

**Figure 3:**
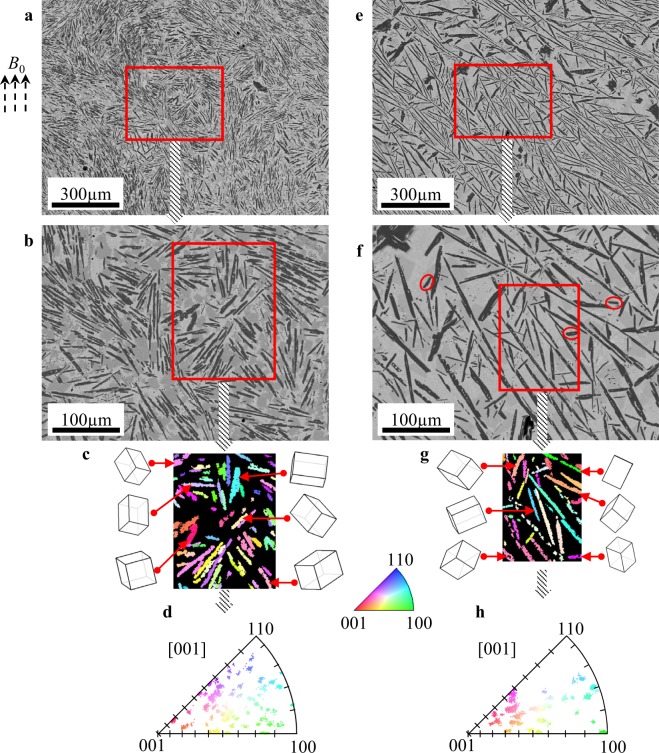
Randomly distributed Nd_2_Fe_14_B platelets when one-step EMV processing was imposed. (**a**) The SEM image shows the structure of the alloy when EMV is imposed at *f* = 250 Hz. (**b**) The magnified SEM image reveals the structure in the red square in (**a**). (**c**) The EBSD map depicts the crystalline orientation of the magnetic phase in red square area in (**b**) beside which space lattices of six crystals are depicted. (**d**) The discrete [001] IPF is generated with the crystals planes being randomly distributed. (**e**) The SEM image shows part of structure of the alloy when EMV is imposed at *f* = 1000 Hz. (**f**) The magnified SEM image reveals the structure in the red square in (**e**). Some isolated fragments are marked by red ellipses. (**g**) The EBSD map of the red square area in (**f**) is shown with space lattices of six crystals with no preference in crystalline orientation distribution. (**h**) The [001] IPF exhibits a similar feature to (**d**) with a random distribution.

The imposition of two-step EMV processing can refine and align magnetic platelets along their *c* axes. The individual role of different frequencies in two-step processing can be clarified when one-step imposition is applied. Figure 3a,b depict the SEM images captured at low and high magnifications at *f* = 250 Hz, showing that the direction of the magnetic platelets is random. Moreover, most of platelets are less than hundred micrometres in their longitude length, indicating that EMV processing at this frequency is effective in segmenting the long compound into short laths. The EBSD map in Fig. 3c is colourful, indicating that crystalline orientations are random, consistent with the space lattices of six crystals coloured from red to blue. No preference in lattice orientation can be inferred, and the corresponding [001] IPF is colourful, as the crystal planes are randomly projected.

When the vibration frequency of one-step EMV processing was set at *f* = 1000 Hz, the partial solidification structure exhibits a similar feature to that observed in the alloy when solidified from the semi-solid state in a static magnetic field without EMV processing (Figs 1b and 3e). Under these two conditions of *f* = 1000 Hz and no EMV, the Nd_2_Fe_14_B phase exhibits long and developed laths. Note that there are a few fine platelets that are isolated within the eutectic area as marked in Fig. 3f., which will be discussed in the later section. The EBSD map is colourful as well, similar to that acquired in Fig. 3c. The space lattices coloured from red to blue show no preference in crystalline orientation, and this is consistent with their [001] IPF in Fig. 3g, in which the projection crystals are randomly distributed.

### Magnetic properties of two alloys

**Figure 4:**
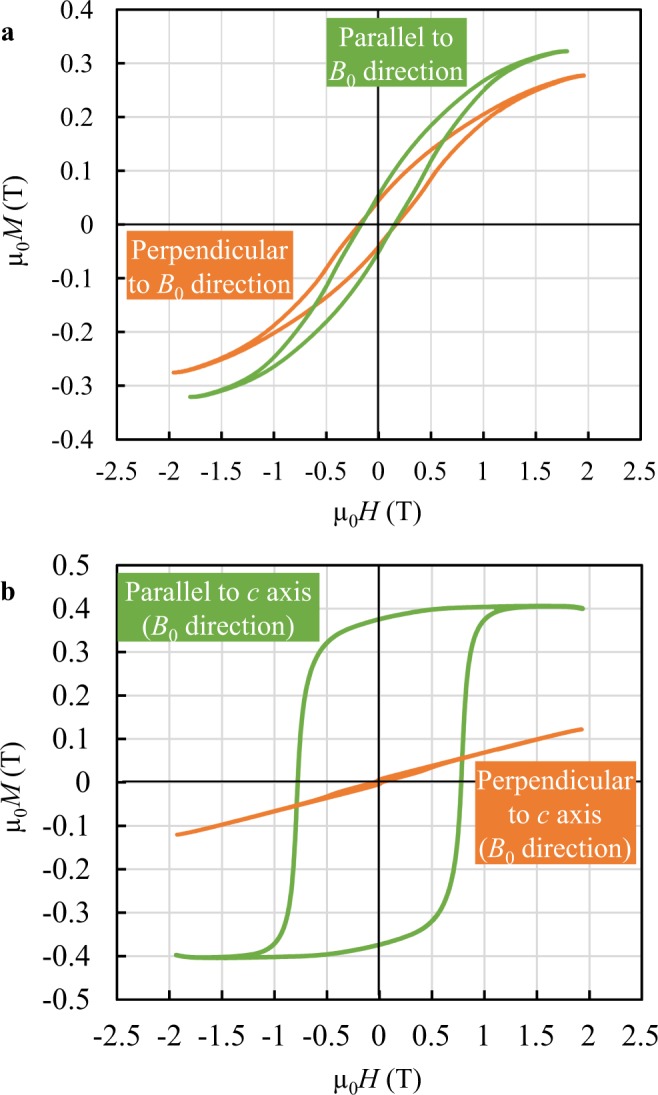
Magnetic hysteresis loops measured in two alloys. (**a**) The alloy was solidified in a static magnetic field without EMV processing. (**b**) The alloy was solidified when the two-step EMV processing was imposed.

As the distribution of the magnetic Nd_2_Fe_14_B phase differs greatly in that it is highly oriented in two-step EMV processing, but randomly distributed without EMV and in one-step EMV processing, it is important to examine the magnetic properties of these two types of alloys. A vibrating sample magnetometer (VSM) was used to characterise the magnetic properties by showing hysteresis loops. Figure 4a depicts the hysteresis loops measured along the parallel and perpendicular directions to the static magnetic field, exhibiting a typical isotropy feature in both loops when the alloy is solidified in the static magnetic field without EMV imposition. The magnetic saturation parallel to the magnetic field is higher than that of the perpendicular direction, which may be ascribed to the alignment of some small Nd_2_Fe_14_B debris in the eutectic liquid, as marked by ellipses in Fig. 3f. In contrast, the saturation remanence and coercivity acquired along the *c* axis of the magnetic platelets are 0.38 T and 0.78 T, respectively, whereas they are almost naught when measured perpendicular to the *c* axis in the alloy that was solidified by two-step EMV processing, as revealed in Fig. 4b, indicating that the present two-step EMV solidification technique is effective in producing a bulk magnet with strong anisotropy in magnetism.

Note that, in comparison with a fully densified Nd_2_Fe_14_B magnet with a volume fraction larger than 90%^9^, the saturation remanence acquired in the present alloy is rather low, as the weight fraction of the magnetic phase is only 30%. Because the saturation remanence of a magnet increases with the weight proportion of the Nd_2_Fe_14_B phase^16^, it is highly expected to achieve higher remanence for the anisotropic magnet that can be applicable in engineering when the fraction of the Nd_2_Fe_14_B compound is increased. Moreover, it has been documented that the refined grain size of Nd_2_Fe_14_B phase is favourable in producing larger coercivity and higher remanence^9^, which motivates to optimise the EMV processing parameters and thus achieve stronger magnets with better properties in future.

## Discussions

Here it is clear that the EMV processing operated at a low frequency of *f* = 250 Hz can segment longer magnetic phase into shorter platelets, whereas it seems that at a high frequency of *f* = 1000 Hz cannot promote the refinement of the magnetic phase, but it is vital in aligning laths once they are segmented into shorter platelets. The present authors have clarified that during EMV processing of the Mg-3wt%Al-1wt%Zn alloy, the primary Mg solid-solution phase will lead the liquid, as the electrical resistivity of the solid is smaller than that of the surrounding liquid^17^, whereas in the Al-1.5 wt%Mn alloy (typically the 3xxx series aluminium alloy)^18^, the primary phase is an intermetallic compound that slugs the counterpart liquid as the electrical resistivity of the intermetallic compound is much larger than that of the remaining liquid. For the present Nd_70_Cu_30_-30wt%Nd_2_Fe_14_B alloy at 973 K, the intermetallic compound phase is solid, and Nd_70_Cu_30_ eutectic is liquid. It is reported^19^ that the electrical resistivity of a Nd_2_Fe_14_B single crystal at room temperature is approximately 1350 nΩ m. Wu *et al*.^20^ found that the electrical resistivity of a bulk Nd_15_Fe_77_B_8_ (near to Nd_2_Fe_14_B in composition) remained constant up to 1000 K, which may be applicable to the present Nd_2_Fe_14_B crystal. According to our knowledge, there have been no reports on the electrical resistivity of Nd_70_Cu_30_ eutectic liquid, which may be estimated from the resistivity of Nd, ca. 650 nΩ m^21^ and Cu, 210 nΩ m,^22^ with a proportion of composition of approximately 518 nΩ m. In this case, the movement of the Nd_2_Fe_14_B compound is to the eutectic liquid what the primary compound in 3xxx Al is to the remaining liquid, i.e., uncoupled movement occurs as the magnetic phase is sluggish while the liquid is mobile.

Quantitatively, the displacement amplitude of phase *i*, s_(*i*)_ = ($\sqrt{2}$*B*_0_ × J)/(4π^3^.*r*^2^.ρ_(*i*)_.*f*^2^) where J is the electric current, *r* is the sample radius, and ρ is the density. The displacement amplitude can be estimated as approximately 1.25 mm and 0.48 mm for the eutectic liquid and magnetic phase at *f* = 250 Hz, respectively. When *f* increases to 1000 Hz, it decreases to ca. 78 µm and 30 µm accordingly. Here it is clear that at *f* = 250 Hz, the leading amplitude of the eutectic liquid is much larger than the length of the feathery magnetic phase. This will generate fluid flow within the semisolid slurry and the compound can be segmented into fine laths as it impedes the movement of the mobile liquid. The severe flow will leave Nd_2_Fe_14_B laths exhibiting a whirl-like pattern in a certain region, as shown in Fig. 3a,b. In contrast, when *f* = 1000 Hz, the leading distance of the eutectic liquid becomes about 50 µm, smaller than the length of the developed magnetic laths and thus the movement may be constrained within the frame, in which some Nd_2_Fe_14_B laths intersect with each other to form a network. Hence, no remarkable segmentation occurs within the network and thus both structures exhibits a similar morphology, as revealed in Figs 1b and 3e.

Before continuing our discussion, we would like to provide a brief explanation of the difference between the widely applied electromagnetic stirring (EMS) technique and the present EMV technique. In both techniques, Lorentz force is generated according to Fleming’s left-hand rule. The direction of the force in EMS is always changing in a rotating magnetic field^23^, whereas it has a constant direction in the static magnetic field, provided that the AC direction remains its sign unchanged while the strength of the force varies with the AC intensity. The imposition of EMV at *f* = 1000 Hz cannot significantly promote the segmentation of the long magnetic phase yielded during mould casting, whereas the application of the directional feature of the Lorentz force enables the eutectic liquid to rotate a refined platelet with its longitude to be parallel to the unidirectional force, by which the resistance from the leading liquid upon the platelet can be minimised. This rotation becomes efficient when the leading amplitude is equivalent to the length of the segmented platelet since; for one aspect, the amplitude should be so small that it must not give rise to macro fluid flow which can randomise the distribution of platelets, and for the other aspect, it should be so large that it can drive a platelet to rotate to minimize its resistance, which is proportional to the projection area of the platelet onto the perpendicular plane of the Lorentz force. Consider that the Nd_2_Fe_14_B phase has been segmented to approximately tens of micrometres after being vibrated at *f* = 250 Hz, the imposition of EMV at *f* = 1000 Hz plays a vital role in rotating the longitude directions to be parallel to the Lorentz force.

As far as the influence of a static magnetic field on solidification is concerned, there have been a great number of reports^24–27^, as Ren *et al*.^27^ briefly reviewed. For the present platelet immersing in the eutectic melt in the static magnetic field, the platelet with its longitude direction parallel to the direction of the Lorentz force does not imply that the *c* axis of the platelet can be aligned to a certain direction, some of them may be parallel while others may have certain degrees with the magnetic field. The anisotropic magnetisation energy, E = *V*(*χ*_*c*_ − *χ*_*a*_)$\,{B}_{0}^{2}$ is yielded in the magnetic field^28^, with *V* being the volume of the solid, *χ*_*c*_ and *χ*_*a*_ are the magnetic susceptibilities along the *c* and *a* axes, respectively. This principle serves not only in synthesising anisotropic magnet in powder metallurgy, but also in orienting anisotropic crystals during solidification, including the superconductor YBa_2_Cu_3_O_7_ oxide^29^, SmCo magnets^30^, AZ91D magnesium alloys^31^, and the Nd_2_Fe_14_B compound in a eutectic liquid^32^. This anisotropic magnetisation energy takes into effect in aligning the crystals whose *c* axes have certain degrees with the static magnetic field.

Here, it should be noted that, for the sake of clarity, the rotation and the alignment of segmented platelets at *f* = 1000 Hz were presented first and then the impact of the static magnetic field on the *c* axis followed. These two forces always coexist upon a platelet, i.e., the rotation and the alignment due to the EMV processing and the impact due to the static magnetic field proceed simultaneously until the *c* axis of the platelet is well aligned, under which condition the platelet becomes energetically minimum and thermodynamically stable.

Finally, it should be noted that, in addition to the present alloy, this technique can be applied to other composite systems, e.g., graphene-reinforced metallic or polymer composites, in which the platelet reinforcement phase may be homogenised to distribute in the matrix at a suitably low EMV frequency and then is well aligned to a certain crystalline orientation at a high vibration frequency. Hence, a composite with a certain anisotropy, e.g., thermal conductivity or electrical conductivity, can be fabricated as the reinforcement phase and the matrix have different electrical properties, which is highly desired in tailoring smart devices in engineering^33^.

## Conclusions

In this study, we used a two-step EMV technique to solidify the Nd_70_Cu_30_-30wt% Nd_2_Fe_14_B alloy from the semi-solid state with two different frequencies. The developed Nd_2_Fe_14_B compound solidified by metallic mould casting can be segmented into short platelets at a low EMV frequency of *f* = 250 Hz, and then these platelets are rotated and aligned at a high EMV frequency of *f* = 1000 Hz with their *c* axes highly oriented parallel to the static magnetic field. The EBSD analysis demonstrated that crystalline orientation of the platelets could be well controlled by the technique. Strong anisotropy in magnetism was verified from the magnetic hysteresis loops, indicating that the present two-step EMV technique is applicable in fabricating strong anisotropic magnets for use in engineering provided that the weight fraction of the Nd_2_Fe_14_B phase in the alloy is increased, and the overall processing is further tuned. The suitable two-step EMV processing enables the production of an anisotropic Nd_2_Fe_14_B magnet via solidification without conventional powder metallurgy routines. Moreover, this technique is highly expected to align the platelet-reinforcement phase in composites, e.g., graphene-reinforced metallic and/or polymer composites that will exhibit remarkable anisotropy, making a technological leap forward in tailoring smart devices.

## Experimental Methods

### Casting ingot preparation

Bulk metals of Fe, Cu (purity better than 99.99%), Nd (purity 99.9%), and B (purity 99.5%) with the nominal composition of Nd_70_Cu_30_-30wt% Nd_2_Fe_14_B were charged into a ceramic crucible and then mounted in a high-frequency induction furnace. The chamber of the furnace was evacuated and then backfilled with inert He gas with a purity greater than 99.9999%. The metals were melted, homogenised, and then cast into a metallic mould with a round cavity of 6 mm in diameter, and thus a cylindrical ingot was prepared.

### EMV experiment

The ingot was machined into a rod of  20 mm in length and then encapsulated into an Al_2_O_3_ tube with the same inner diameter as the ingot. The ingot was fixed by two carbon blocks that were connected with Cu electrodes so that alternating current could flow. A blind hole was made in the wall of the Al_2_O_3_ tube, in which a K-type thermocouple was embedded to monitor the temperature of the specimen. A movable arc-shaped carbon heater covered the specimen and then the setting kit was placed into a bore with the static magnetic flux density of *B*_0_ = 10 T. Further details on the experimental setup could be found elsewhere^15^. The specimen was heated to 973 K, i.e., 180 K higher than the melting point of CuNd-Nd eutectics (793 K) and approximately 480 K lower than the peritectic decomposition temperature of the Nd_2_Fe_14_B compound (1453 K), resulting in a semisolid state of the specimen. The sample was kept at this temperature for 120 s to homogenise the temperature distribution. For comparison, the sample was first solidified from the semi-solid state in the static magnetic field only, without the imposition of alternating current (AC). For the EMV experiment, AC was switched on to vibrate the sample at *f* = 250 Hz for 120 s, then the AC frequency was increased to 1000 Hz for another 120 s, and finally both the vibration power and the heating power of the carbon heater were switched off simultaneously to cool the sample. This operation was termed two-step EMV processing. To clarify the effect of the respective vibration frequency on structure formation, one-step EMV, i.e., only 250 Hz was imposed for 120 s or 1000 Hz was imposed for 120 s upon an individual sample, was accomplished after the sample temperature was homogenised at 973 K. This operation was termed one-step EMV processing. After the sample was solidified, a notch was made along the longitudinal direction in the location where the thermocouple was set just beneath the Al_2_O_3_ tube. This was important in revealing the orientation distribution of magnetic phase.

### Microstructure observation

The sample after solidification was heated treated to 623 K, i.e., approximately 36 K higher than the Curie temperature (587 K) of the magnetic phase, for 30 min to demagnetise in the vacuum condition. All samples in the present study were sliced with the sectioning plane parallel to the diameter direction, and thus were also parallel to the direction of the static magnetic field except for Fig. 2f that was cut perpendicular to the direction of the static magnetic field. The sample was mounted, ground, polished, and observed under an optical microscope and a scanning electron microscope (SEM). The SEM image was captured under backscattered electron (BSE) mode to reveal the composition. The energy-dispersive X-ray spectroscopy (EDS) analysis of a macro area (2.5 mm × 2 mm) showed that the overall composition of samples prepared either by metallic mould casting or after EMV processing, e.g., Nd:Fe:Cu = 67.3:22.5:10.2 (mass ratio, hereinafter), was almost identical with that of theoretical value of the Nd_70_Cu_30_-30wt% Nd_2_Fe_14_B alloy (Nd:Fe:Cu = 67:21.9:11.1). Spot EDS analysis of black phases revealed that the ratio of Nd and Fe was ca. 69:31, near to the theoretical value in Nd_2_Fe_14_B phase being of 73:27 when considering the measurement error of the technique. This indicated that the black phase was Nd_2_Fe_14_B phase in the BSE mode, whereas it was white in the optical macro view because of light reflection. The mass ratio of Nd and Cu of the grey phase was consistent with that of CuNd-Nd eutectics, exhibiting from grey to light grey in the BSE mode in the present observation.

### Crystalline orientation characterization

For the EBSD observation, after the sample was ground, it was finely polished using silica colloidal that was essential to generate Kikuchi patterns. As the present study focused on the orientation of Nd_2_Fe_14_B platelets, only the magnetic phase was indexed and highlighted in decoding Kikuchi patterns. The [001] inverse pole figure (IPF) was generated based on the EBSD map, in which the misorientation angles between neighbouring bars in the scale were 10 degrees. Space lattices of typical crystals were generated to show the evidence of crystalline orientation after solidification.

### Magnetic properties measurement

The sliced specimen was delicately fixed into a holder according to the position of the notch and the arrangement direction of the Nd_2_Fe_14_B platelets. The holder was then set in a vibrating sample magnetometer (VSM). For the sample solidified without EMV in the static magnetic field, magnetic hysteresis loops were achieved when the magnetic field of the VSM was imposed perpendicularly and parallelly to the static magnetic field. For the sample solidified subject to two-step EMV processing, the magnetic hysteresis loops were obtained when the magnetic field of the VSM was perpendicular and parallel to the *c* axis of the well oriented magnetic platelets, which corresponds to the perpendicular and parallel direction of the static magnetic field *B*_0_ during EMV processing. A high-purity Ni (greater than 99.99%) slice with almost the same specimen specification (mass difference was less than 1%) was used to calibrate the raw data under the same measurement condition. Sample density was required for calibration, which was measured using Archimedes’ principle in ethanol and found to be 7.311 g/cm^3^.

## Data Availability

The data that support the findings of this study are available from the corresponding author on reasonable request.
